# Springer: An R package for bi-level variable selection of high-dimensional longitudinal data

**DOI:** 10.3389/fgene.2023.1088223

**Published:** 2023-04-06

**Authors:** Fei Zhou, Yuwen Liu, Jie Ren, Weiqun Wang, Cen Wu

**Affiliations:** ^1^ Department of Statistics, Kansas State University, Manhattan, KS, United States; ^2^ Department of Biostatistics and Health Data Sciences, Indiana University School of Medicine, Indianapolis, IN, United States; ^3^ Department of Food, Nutrition, Dietetics and Health, Kansas State University, Manhattan, KS, United States

**Keywords:** bi-level variable selection, gene–environment interaction, repeated measurements, generalized estimating equation, quadratic inference function

## Abstract

In high-dimensional data analysis, the bi-level (or the sparse group) variable selection can simultaneously conduct penalization on the group level and within groups, which has been developed for continuous, binary, and survival responses in the literature. Zhou et al. (2022) (PMID: 35766061) has further extended it under the longitudinal response by proposing a quadratic inference function-based penalization method in gene–environment interaction studies. This study introduces “springer,” an R package implementing the bi-level variable selection within the QIF framework developed in Zhou et al. (2022). In addition, R package “springer” has also implemented the generalized estimating equation-based sparse group penalization method. Alternative methods focusing only on the group level or individual level have also been provided by the package. In this study, we have systematically introduced the longitudinal penalization methods implemented in the “springer” package. We demonstrate the usage of the core and supporting functions, which is followed by the numerical examples and discussions. R package “springer” is available at https://cran.r-project.org/package=springer.

## 1 Introduction

In gene–environment interaction studies, a central task is to detect important G×E interactions that are beyond main G and E effects. Although the main environmental factors are usually preselected and of low dimensionality, in the presence of a large number of G factors, conducting G×E analysis can be performed in the variable selection framework. Recently, Zhou et al. (2021a) surveyed the penalized variable selection methods for interaction analysis, revealing the pivotal role that the sparse group selection played in G×E studies. Specifically, determining whether a genetic factor, such as the gene expression or SNP, is associated with the disease phenotype is equivalent to feature selection on the group level of main G and G×E interactions with respect to that G factor. Further detection of the main and/or interaction effects demands selection within the group. Such bi-level variable selection methods have been extensively studies under continuous, binary, and survival outcomes in G×E studies (Wu et al., 2018a; Ren et al., 2022a; Ren et al., 2022b; Liu et al., 2022).

Zhou et al. (2022a) have further examined the sparse group variable selection for longitudinal studies where measurements on the subjects are repeatedly recorded over a sequence of units, such as time (Verbeke et al., 2014). In general, major competitors for the bi-level selection include LASSO and group LASSO types of regularization methods that only perform variable selection on the individual and group levels, respectively (Wu and Ma, 2015). Zhou et al. (2022a) have also incorporated two alternatives for comparison under the longitudinal response based on the quadratic inference functions (QIFs) (Qu et al., 2000). The sgQIF, gQIF, and iQIF, denoting the penalized QIF methods accommodating sparse group, group-, and individual-level selections, respectively, have been thoroughly examined with different working correlation structures modeling the relatedness among repeated measurements. All these methods have been implemented in R package *springer*.

In this article, we provide a detailed introduction of R package *springer*, which has implemented not only the proposed and alternative regularized QIF methods from Zhou et al. (2022a) but also their counterparts based on the generalized estimating equations (GEEs) (Liang and Zeger, 1986). The GEE, originally proposed by Liang and Zeger (1986), captures the intra-correlation of repeated measurements using their marginal distributions and a working correlation matrix depending on certain nuisance parameters. The QIF has further improved upon GEE *via* bypassing the nuisance parameters, leading to consistent and optimal estimation of regression coefficients even when the working correlation is misspecified (Qu et al., 2000).

GEE and QIF have been the two major frameworks for developing high-dimensional penalization methods, especially under the main effect models. For example, Wang et al. (2012) have proposed a regularized GEE with the SCAD penalty. Cho and Qu (2013) have considered the penalized QIF with penalty functions including LASSO, adaptive LASSO, and SCAD. More recently, the high-dimensional longitudinal interaction models have been developed based on GEE and QIF (Zhou et al., 2019; Zhou et al., 2022a). In terms of statistical software, R package *PGEE*, developed by Inan and Wang (2017), has implemented the penalized GEE methods from Wang et al. (2012). The package *interep* features the mixture of individual- and group-level penalty under the GEE, where selection on the two levels does not overlap and thus is not a sparse group penalty (Zhou et al., 2019; Zhou et al., 2022b).

Package *springer* is among the first of statistical software to systematically implement bi-level, group-level, and individual-level regularization under both GEE and QIF. It focuses on the longitudinal interaction models where the linear G×E interactions have been assumed (Zhou et al., 2021a). The non-linear G×E interactions usually demand the varying coefficient models and their extensions (Wu and Cui, 2013; Wu et al., 2018b; Ren et al., 2020). In longitudinal studies, Wang et al. (2008) and Tang et al. (2013) have developed regularized variable selection based on varying coefficient (VC) models under the least squares and quantile check loss, respectively. They have assumed independence for repeated measurements, so the within-subject correlation has not been incorporated. Chu et al. (2016), on the other hand, have considered the weighted least squares-based VC models, where the weights have been estimated from a marginal non-parametric model to account for intra-cluster interconnections. R package *VariableScreening* has provided the corresponding R codes and examples.

We have made R package *springer* publicly available on CRAN (Zhou et al., 2021b). The core modules of the package have been developed in C++ for fast computation. We organize the rest of the paper as follows. Section 2 provides a summary of bi-level penalization in longitudinal interaction studies. The main and supporting functions in package *springer* are introduced in Section 3. To demonstrate the usage of the package, we present a simulated example in Section 4 and a case study in Section 5. We conclude the article with discussions in Section 6.

## 2 Materials and methods

### 2.1 The bi-level model for longitudinal G×E studies

In a typical longitudinal setting with *n* subjects, the *i*th subject (1⩽*i*⩽*n*) is repeatedly measured over *t*_*i*_ time points, which naturally results in *t*_*i*_ repeated measurements that are correlated for the same subject and are assumed to be independent with the measurements taken from other subjects. Then, *Y*_*ij*_ denotes the phenotype measured for the *i*th subject at time point *j* (1⩽*j*⩽*t*_*i*_). $G_{ij}={\left(G_{\mathrm{ij1}},\ldots ,G_{\mathrm{ijp}}\right)}^{⊤}$ and $E_{ij}={\left(E_{\mathrm{ij1}},\ldots ,E_{\mathrm{ijq}}\right)}^{⊤}$ represent the *p*-dimensional vector of genetic factors and the *q*-dimensional vector of environmental factors, respectively. The bi-level G×E model associates the genetic and environmental main effects and their interactions with the repeatedly measured phenotypic response as follows:

$$\begin{array}{cc} Y_{ij} & =\mu_{ij}+\epsilon_{ij}\\ & =\alpha_{n0}+\overset{q}{\underset{h=1}{\sum }}\alpha_{nh}E_{\mathrm{ijh}}+\overset{p}{\underset{k=1}{\sum }}\gamma_{nk}G_{\mathrm{ijk}}+\overset{p}{\underset{k=1}{\sum }}\overset{q}{\underset{h=1}{\sum }}u_{\mathrm{nhk}}E_{\mathrm{ijh}}G_{\mathrm{ijk}}+\epsilon_{ij}\\ & =\alpha_{n0}+\overset{q}{\underset{h=1}{\sum }}\alpha_{nh}E_{\mathrm{ijh}}+\overset{p}{\underset{k=1}{\sum }}\left(\gamma_{nk}+\overset{q}{\underset{h=1}{\sum }}u_{\mathrm{nhk}}E_{\mathrm{ijh}}\right)G_{\mathrm{ijk}}+\epsilon_{ij}\\ & =\alpha_{n0}+\overset{q}{\underset{h=1}{\sum }}\alpha_{nh}E_{\mathrm{ijh}}+\overset{p}{\underset{k=1}{\sum }}\eta_{nk}^{⊤}Z_{\mathrm{ijk}}+\epsilon_{ij}, \end{array}$$ (1)

where *α*_*n*0_ is the intercept, and *α*_*nh*_, *γ*_*nk*_, and *u*_*nhk*_ denote the regression coefficients of environmental and genetic main effects and their interactions, correspondingly. We also define $\eta_{nk}={\left(\gamma_{nk},u_{\mathrm{n1k}},\ldots ,u_{\mathrm{nqk}}\right)}^{⊤}$ , and $Z_{\mathrm{ijk}}={\left(G_{\mathrm{ijk}},E_{\mathrm{ij1}}G_{\mathrm{ijk}},\ldots ,E_{\mathrm{ijq}}G_{\mathrm{ijk}}\right)}^{⊤}$ . *Z*_*ijk*_ is a (*q* + 1)-dimensional vector representing the main and interaction effects with respect to the *k*th genetic factor. For 1⩽*j*⩽*t*_*i*_, the random error *ϵ*_*ij*_ has mean zero and a finite variance. For convenience, the random error *ϵ*_*i*_ is assumed to be multivariate normal as $\epsilon_{i}={\left(\epsilon_{i1},\ldots ,\epsilon_{it_{i}}\right)}^{⊤}∼N_{t_{i}}\left(0,\Sigma_{i}\right)$ , where Σ_*i*_ is the covariance matrix corresponding to the *i*th subject. From now on, we let *t*_*i*_ = *t*. Combined, we can write $\alpha_{n}={\left(\alpha_{n1},\ldots ,\alpha_{nq}\right)}^{⊤}$ , $\eta_{n}={\left(\eta_{n1}^{⊤},\ldots ,\eta_{np}^{⊤}\right)}^{⊤}$ , and $Z_{ij}={\left(Z_{\mathrm{ij1}}^{⊤},\ldots ,Z_{\mathrm{ijp}}^{⊤}\right)}^{⊤}$ . The length of the coefficient vector *η*_*n*_ is *p* + *pq*. Then, model (1) can be equivalently expressed as

$$Y_{ij}=\alpha_{n0}+E_{ij}^{⊤}\alpha_{n}+Z_{ij}^{⊤}\eta_{n}+\epsilon_{ij}.$$

The (1 + *q* + *p* + *pq*)-dimensional vectors $\beta_{n}={\left(\alpha_{n0},\alpha_{n}^{⊤},\eta_{n}^{⊤}\right)}^{⊤}$ and $W_{ij}={\left(1,E_{ij}^{⊤},Z_{ij}^{⊤}\right)}^{⊤}$ are denoted, and a concise form of model (1) is formed as follows:

$$Y_{ij}=W_{ij}^{⊤}\beta_{n}+\epsilon_{ij}.$$

The aforementioned model provides a general formulation under the longitudinal design in which both the response variable and predictors are repeatedly measured. Here, the predictors are G and E main effects and G×E interactions. It still works when only one or neither of the G and E factors are repeatedly measured. In the real data analyzed in Zhou et al. (2022a), both the G and E factors in the interaction study do not vary across time.

### 2.2 An overview of interaction studies based on GEE and QIF

R package *springer* (Zhou et al., 2021b) includes methods that account for repeated measurements based on the GEE and QIF, respectively. Here, we briefly review the two frameworks for longitudinal interaction studies.

The **generalized estimating equation** has been proposed by Liang and Zeger (1986) to account for intra-cluster correlations using a marginal model by specifying the conditional expectation and variance of each response, *Y*_*ij*_, and the conditional pairwise within-subject association among the vector of repeatedly measured phenotypes. In the longitudinal interaction studies, the marginal expectation of the response is $\text{E}\left(Y_{ij}\right)=\mu_{ij}=W_{ij}^{T}\beta_{n}$ , and the conditional variance of *Y*_*ij*_ is Var(*Y*_*ij*_) = *δ*(*μ*_*ij*_), where *δ*(*μ*_*ij*_) is a known function of the mean *μ*_*ij*_. Then, the score equation for the longitudinal G×E model is defined as

$$\overset{n}{\underset{i=1}{\sum }}\frac{\partial \mu_{i}\left(\beta_{n}\right)}{\partial \beta_{n}}V_{i}^{-1}\left(Y_{i}-\mu_{i}\left(\beta_{n}\right)\right)=0,$$

where $Y_{i}={\left(Y_{i1},\ldots ,Y_{it}\right)}^{⊤}$ and the covariance matrix for the intra-subject association *V*_*i*_ is defined as $V_{i}=A_{i}^{\frac{1}{2}}R_{i}\left(\nu \right)A_{i}^{\frac{1}{2}}$ . Here, for the *i*th subject, the diagonal matrix *A*_*i*_ is defined as *A*_*i*_ = diag{Var(*Y*_*i*1_), …, Var(*Y*_*it*_)}, and the “working” correlation matrix *R*_*i*_(*ν*) depends on a finite dimensional parameter vector *ν*, characterizing the within-subject association. We have $\mu_{i}\left(\beta_{n}\right)={\left(\mu_{i1}\left(\beta_{n}\right),\ldots ,\mu_{it}\left(\beta_{n}\right)\right)}^{⊤}$ . The ratio term in the aforementioned score equation is equivalent to $W_{i}={\left(W_{i1},\ldots ,W_{it}\right)}^{⊤}$ . Then, the GEE estimator, $\hat{\beta_{n}}$ , is the corresponding solution.

The term “working” correlation in GEE is adopted to distinguish *R*_*i*_(*ν*) from the true underlying correlation among intra-subject measurements. Liang and Zeger (1986) have shown that when *ν* is consistently estimated, the GEE estimator is consistent even if the correlation structure is not correctly specified. However, there is a cost under such misspecification, that is, the GEE estimator is no longer efficient, and *ν* cannot be consistently estimated.

The **quadratic inference function** overcomes the disadvantage of GEE by avoiding the direct estimation of *ν* (Qu et al., 2000). It has also been shown that even when the correlation structure is misspecified, the QIF estimator is still optimal. With the bi-level modeling of G×E interactions under the longitudinal response, the inverse of *R*(*ν*) can be calculated by a linear combination of basis matrices within the QIF framework. Specifically, $\text{R}{\left(\nu \right)}^{-1}\approx \sum_{k=1}^{m}c_{k}B_{k}$ , where *B*_1_ is an identity matrix and *B*_2_, … , *B*_*m*_ are symmetric basis matrices with unknown coefficients *c*_1_, … *c*_*m*_. The specifications of these basis matrices are dependent on the types of working correlation (Qu et al., 2000). The score equations can be rewritten as

$$\overset{n}{\underset{i=1}{\sum }}W_{i}^{⊤}A_{i}^{-\frac{1}{2}}\left(c_{1}B_{1}+\cdots +c_{m}B_{m}\right)A_{i}^{-\frac{1}{2}}\left(Y_{i}-\mu_{i}\left(\beta_{n}\right)\right).$$ (2)

Accordingly, for the *i*th subject, we define the extended score vector, *ϕ*_*i*_ (*β*_*n*_), for the bi-level G×E model as

$$\phi_{i}\left(\beta_{n}\right)=\left(\begin{array}{c} W_{i}^{⊤}A_{i}^{-\frac{1}{2}}B_{1}A_{i}^{-\frac{1}{2}}\left(Y_{i}-\mu_{i}\left(\beta_{n}\right)\right)\\ .\\ .\\ .\\ W_{i}^{⊤}A_{i}^{-\frac{1}{2}}B_{m}A_{i}^{-\frac{1}{2}}\left(Y_{i}-\mu_{i}\left(\beta_{n}\right)\right) \end{array}\right).$$ (3)

We then denote the extended score for all subjects as $\bar{\phi_{n}}\left(\beta_{n}\right)=\frac{1}{n}\sum_{i=1}^{n}\phi_{i}\left(\beta_{n}\right)$ . The linear combination of all components in $\bar{\phi_{n}}\left(\beta_{n}\right)$ directly leads to the estimation functions in Eq. 2. The quadratic inference function based on the extended score $\bar{\phi_{n}}\left(\beta_{n}\right)$ is defined as

$$Q_{n}\left(\beta_{n}\right)={\bar{\phi_{n}}}^{⊤}\left(\beta_{n}\right)\bar{\Omega_{n}}{\left(\beta_{n}\right)}^{-1}\bar{\phi_{n}}\left(\beta_{n}\right),$$

where the sample covariance matrix of *ϕ*_*i*_ (*β*_*n*_) is $\bar{\Omega_{n}}\left(\beta_{n}\right)=\frac{1}{n}\sum_{i=1}^{n}\phi_{i}\left(\beta_{n}\right)\phi_{i}{\left(\beta_{n}\right)}^{⊤}$ . Minimizing the aforementioned quadratic inference function yields $\hat{\beta_{n}}$ , i.e., $\hat{\beta_{n}}=\text{arg}\underset{\beta_{n}}{\text{min}}Q_{n}\left(\beta_{n}\right)$ . It should be noted that the minimization does not involve the coefficients *c*_1_, … *c*_*m*_ in Eq. 2.

### 2.3 Penalized QIF for the bi-level longitudinal G×E interaction studies

R package *springer* (Zhou et al., 2021b) can perform penalized sparse group variable selection based on both the GEE and QIF framework in order to identify an important subset of main and interaction effects that are associated with the longitudinal phenotype. As QIF is an extension of GEE, we focus on the penalized bi-level QIF in the main text and introduce GEE-based methods in the Supplementary Appendix. The following regularized bi-level QIF has been proposed in Zhou et al. (2022a):

$$U\left(\beta_{n}\right)=Q\left(\beta_{n}\right)+\overset{p}{\underset{k=1}{\sum }}\rho \left(\|\eta_{nk}{\|}_{\Sigma_{k}};\lambda_{1},\gamma \right)+\overset{p}{\underset{k=1}{\sum }}\overset{q+1}{\underset{h=1}{\sum }}\rho \left(|\eta_{\mathrm{nkh}}|;\lambda_{2},\gamma \right),$$ (4)

where the minimax concave penalty is $\rho \left(t;\lambda ,\gamma \right)=\lambda \int_{0}^{t}{\left(1-\frac{x}{\gamma \lambda }\right)}_{+}dx$ on [0, *∞*) with the tuning parameter *λ* and regularization parameter *γ* (Zhang, 2010). The group-level penalty $\rho \left(\|\eta_{nk}{\|}_{\Sigma_{k}};\lambda_{1},\gamma \right)$ is imposed on $\|\eta_{nk}{\|}_{\Sigma_{k}}$ , which is the empirical norm of *η*_*nk*_, to determine whether the *k*th SNP has any contribution to the variation in the repeatedly measured phenotype. We define the empirical norm as $\|\eta_{nk}{\|}_{\Sigma_{k}}={\left(\eta_{nk}\Sigma_{k}\eta_{nk}\right)}^{1/2}$ with $\Sigma_{k}=n^{-1}B_{k}^{⊤}B_{k}$ , where *B*_*k*_ is the subset of the design matrix corresponding to the interactions between the *k*th genetic factor and all the E factors. If *η*_*nk*_ is estimated as a zero vector, the *k*th SNP is not associated with the phenotypic response. Otherwise, the individual-level penalty *ρ*(|*η*_*nkh*_|; *λ*_2_, *γ*) further selects the main and interaction effects that are associated with the phenotype.

Our choice of the baseline penalty function is the MCP, and the corresponding first derivative function of MCP is defined as $\rho^{\prime }\left(t;\lambda ,\gamma \right)=\left(\lambda -\frac{t}{\gamma }\right)I\left(0\leq t\leq \gamma \lambda \right)$ .

The penalized QIF in (4) is the extension of bi-level variable selection to longitudinal studies, which conducts selections of important groups and individual members within the group simultaneously. It is worth noting that the penalized GEE model proposed by Zhou et al. (2019) does not perform within-group selection. The shrinkage has been imposed on the individual level (G main effect) and group level (G×E interactions) separately. Unlike the model in (4), the terms selected on the individual level in the study by Zhou et al. (2019) are not members of the group. Therefore, it is not the sparse group selection, although in a loose sense, it can be treated as a bi-level variable selection method.

A general form for the objective function of regularization methods is “unpenalized objective function + penalty function” (Wu and Ma, 2015). QIF and GEE are widely adopted unregularized objective functions for repeated measurement studies. LASSO and SCAD have been considered the penalty functions in longitudinal studies, where selection of the main effects are of interest (Wang et al., 2012; Cho and Qu, 2013; Ma et al., 2013). To accommodate more complicated structured sparsity incurred by interaction effects, the shrinkage components in Eq. 4 adopts MCP as the baseline penalty to perform individual- and group-level penalization simultaneously. It is commonly recognized that the structure-specific regularization functions are needed to accommodate different sparsity patterns. For example, to account for strong correlations among predictors, network-based variable selection methods have been developed (Ren et al., 2019; Huang et al., 2021). The penalty functions have been implemented in a diversity of R packages. For example, under generalized linear models, the package *glmnet* has included LASSO and its extensions, such as the ridge penalty and elastic net (Friedman et al., 2010a). R package *regnet* has been developed for network-based penalization under continuous, binary, and survival responses with possible choices on robustness (Ren et al., 2017; Ren et al., 2019). With the longitudinal response, R package *PGEE* has adopted SCAD penalty for penalized GEE to select main effects (Inan and Wang, 2017), and package *interep* has been designed in interaction studies based on MCP (Zhou et al., 2022b).

### 2.4 The bi-level selection algorithm based on QIF

Optimization of the penalized QIF in (4) demands the Newton–Raphson algorithm that can update ${\hat{\beta }}_{n}$ iteratively. Specifically, the estimated coefficient vector ${\hat{\beta }}_{n}^{g+1}$ can be obtained based on ${\hat{\beta }}_{n}^{g}$ at the *g*th iteration as follows:

$${\hat{\beta }}_{n}^{g+1}={\hat{\beta }}_{n}^{g}+{\left[V\left({\hat{\beta }}_{n}^{g}\right)+nH\left({\hat{\beta }}_{n}^{g}\right)\right]}^{-1}\left[P\left({\hat{\beta }}_{n}^{g}\right)-nH\left({\hat{\beta }}_{n}^{g}\right){\hat{\beta }}_{n}^{g}\right],$$ (5)

where $P\left({\hat{\beta }}_{n}^{g}\right)$ and $V\left({\hat{\beta }}_{n}^{g}\right)$ can be obtained as

$$P\left({\hat{\beta }}_{n}^{g}\right)=-\frac{\partial Q\left({\hat{\beta }}_{n}^{g}\right)}{\partial \beta_{n}}=-2\frac{\partial {\bar{\phi_{n}}}^{⊤}}{\partial \beta_{n}}{\bar{\Omega_{n}}}^{-1}\bar{\phi_{n}}\left({\hat{\beta }}_{n}^{g}\right),$$

and

$$V\left({\hat{\beta }}_{n}^{g}\right)=\frac{\partial^{2}Q\left({\hat{\beta }}_{n}^{g}\right)}{\partial^{2}\beta_{n}}=2\frac{\partial {\bar{\phi_{n}}}^{⊤}}{\partial \beta_{n}}{\bar{\Omega_{n}}}^{-1}\frac{\partial \bar{\phi_{n}}}{\partial \beta_{n}}.$$

Moreover, $H\left({\hat{\beta }}_{n}^{g}\right)$ is a diagonal matrix consisting of derivatives of both the individual-and group-level penalty functions, which is defined as

$$\begin{array}{cc} H\left({\hat{\beta }}_{n}^{g}\right) & =\text{diag}(\left(\underset{1+q}{\underbrace{0,\ldots ,0}},\underset{1+q}{\underbrace{\frac{\rho^{\prime }\left(\|{\hat{\eta }}_{n1}^{g}{\|}_{\Sigma_{1}};\sqrt{q+1}\lambda_{1},\gamma \right)}{\epsilon +\|{\hat{\eta }}_{n1}^{g}{\|}_{\Sigma_{1}}},\ldots ,\frac{\rho^{\prime }\left(\|{\hat{\eta }}_{n1}^{g}{\|}_{\Sigma_{1}};\sqrt{q+1}\lambda_{1},\gamma \right)}{\epsilon +\|{\hat{\eta }}_{n1}^{g}{\|}_{\Sigma_{1}}}}},\ldots ,\right.\\ & \left.\underset{1+q}{\underbrace{\frac{\rho^{\prime }\left(\|{\hat{\eta }}_{np}^{g}{\|}_{\Sigma_{p}};\sqrt{q+1}\lambda_{1},\gamma \right)}{\epsilon +\|{\hat{\eta }}_{np}^{g}{\|}_{\Sigma_{p}}},\ldots ,\frac{\rho^{\prime }\left(\|{\hat{\eta }}_{np}^{g}{\|}_{\Sigma_{p}};\sqrt{q+1}\lambda_{1},\gamma \right)}{\epsilon +\|{\hat{\eta }}_{np}^{g}{\|}_{\Sigma_{p}}}}})\right)+\text{diag}(\left(\underset{1+q}{\underbrace{0,\ldots ,0}},\right.\\ & \left.\underset{1+q}{\underbrace{\frac{\rho^{\prime }\left(|{\hat{\eta }}_{\mathrm{n11}}^{g}|;\lambda_{2},\gamma \right)}{\epsilon +|{\hat{\eta }}_{\mathrm{n11}}^{g}|},\ldots ,\frac{\rho^{\prime }\left(|{\hat{\eta }}_{n1\left(q+1\right)}^{g}|;\lambda_{2},\gamma \right)}{\epsilon +|{\hat{\eta }}_{n1\left(q+1\right)}^{g}|}}},\ldots ,\underset{1+q}{\underbrace{\frac{\rho^{\prime }\left(|{\hat{\eta }}_{\mathrm{np1}}^{g}|;\lambda_{2},\gamma \right)}{\epsilon +|{\hat{\eta }}_{\mathrm{np1}}^{g}|},\ldots ,\frac{\rho^{\prime }\left(|{\hat{\eta }}_{np\left(q+1\right)}^{g}|;\lambda_{2},\gamma \right)}{\epsilon +|{\hat{\eta }}_{np\left(q+1\right)}^{g}|}}}\right)), \end{array}$$

where the small positive fraction *ϵ* is set to 10^–6^ to guarantee the numerical stability when the denominator approaches zero. Since the intercept and the environmental factors are not subject to shrinkage selection, the first (1 + *q*) entries on the main diagonal of the matrix are zero accordingly. With fixed tuning parameters, ${\hat{\beta }}_{n}^{g+1}$ is updated iteratively following Eq. 5. The update stops when the convergence criterion has been reached, that is, the difference between the L_1_ norm of ${\hat{\beta }}_{n}^{g+1}$ and ${\hat{\beta }}_{n}^{g}$ is less than a cutoff (e.g., 0.001). Numerical studies have shown that only a small to moderate number of iterations are required upon convergence (Zhou et al., 2022a).

The sparse group penalty (4) incorporates two tuning parameters, *λ*_1_ and *λ*_2_, to determine the amount of shrinkage on the group and individual level, correspondingly. An additional regularization parameter *γ* further balances the unbiasedness and convexity of MCP. The performance of the proposed regularized QIF is insensitive under different choices of *γ* (Zhou et al., 2022a). The best pair of (*λ*_1_, *λ*_2_) can be searched over the two-dimensional grid through *K*-fold cross-validation. We first split the dataset into *K* non-overlapping portions of roughly the same size and held out the *k*th (*k* = 1, … ,*K*) fold as the testing dataset. The rest of the data are used as training data to fit a regularized QIF by giving a specific pair of (*λ*_1_, *λ*_2_). *n*_*k*_ and *n*_−*k*_ denote the index sets of subjects as training and testing samples, respectively. We can compute the prediction error on testing data as

$${\text{PE}}_{-k}\left(\lambda_{1},\lambda_{2}\right)=\frac{1}{\left|n_{-k}\right|}\underset{i\in n_{-k}}{\sum }{\left(Y_{i}-\mu_{i}\left({\hat{\beta }}_{n_{k}}\right)\right)}^{2},$$

where |*n*_−*k*_| is the size of testing data, and ${\hat{\beta }}_{n_{k}}$ is the regularized coefficient obtained using the training data. The computation cycles through each of the *K* fold for *k* = 1, 2. ., *K*, yielding the following cross-validation error:

$$\text{CV}\left(\lambda_{1},\lambda_{2}\right)=\frac{1}{K}\overset{K}{\underset{k=1}{\sum }}{\text{PE}}_{-k}\left(\lambda_{1},\lambda_{2}\right).$$ (6)

The cross-validation value with respect to each pair of (*λ*_1_, *λ*_2_) can be retrieved across the entire two-dimensional grid. The optimal pair of tunings is corresponding to the smallest CV value. Details of the algorithm are given as follows:

1 The two-dimensional grid of (*λ*_1_, *λ*_2_) is provided with an appropriate range.
2 Under the fixed (*λ*_1_, *λ*_2_),
(a) ${\hat{\beta }}_{n}^{0}$ is initialized using LASSO
(b) at the (*g* + 1)^th^ iteration, $V\left({\hat{\beta }}_{n}^{g}\right),H\left({\hat{\beta }}_{n}^{g}\right),P\left({\hat{\beta }}_{n}^{g}\right)$ is computed and
(c) ${\hat{\beta }}_{n}^{d+1}$ is updated according to Eq. 5.
(d) The cross-validation error is calculated using Eq. 6.
3 Step 2 is repeated for each pair of (*λ*_1_, *λ*_2_) until convergence.
4 The optimal (*λ*_1_, *λ*_2_) is found under the smallest cross-validation error. The corresponding ${\hat{\beta }}_{n}$ is reported.

The validation approach is a popular alternative of tuning selection to bypass the computational intensity of cross-validation. When the data-generating model is available, the independent testing data with much larger size can be readily generated. Then, the prediction performance of the fitted sparse group PQIF model under (*λ*_1_, *λ*_2_) can be assessed on the testing data directly. On the contrary, in cross-validation, the prediction error can only be obtained after cycling through all the *K* folds as shown by Equation 6.

## 3 R package *springer*

Package *springer* includes two core functions, namely, springer and cv.springer. The function springer can fit both GEE- and QIF-based penalization models under longitudinal responses in G×E interaction studies. The function cv.springer computes the prediction error in cross-validation. Moreover, the package also includes supporting functions reformat, penalty, and dmcp, which have been developed by the authors. To speed up computation, we have implemented the Newton–Raphson algorithms in C++. The package is thus dependent on R packages Rcpp and RcppArmadillo (Eddelbuettel and François, 2011; Eddelbuettel, 2013; Eddelbuettel and Sanderson, 2014).

### 3.1 The core functions

In package *springer*, the R function for computing the penalized estimates under fixed tuning parameters is

springer (clin = NULL,e, g, y, beta0, func, corr, **structure**, lam1, lam2, maxits = 30,tol = 0.001).

The clinical covariates and environmental and genetic factors can be specified by the input arguments clin, e, and g, respectively. This is different from packages conducting feature selection for the main effects, such as *glmnet* and *PGEE*, where the entire design matrix should be used an input (Friedman et al., 2010a; Inan and Wang, 2017). In interaction studies, the design matrix has a much more complicated structure. Our package is user friendly in that users only need to provide the clinical, g, and e factors, and then the function springer will automatically formulate the design matrix tailored for interaction analysis. The clinical covariates are not involved in the interactions with G factors and are not subject to selection. The argument beta0 denotes the initial value of ${\hat{\beta }}_{n}^{0}$ , which is used at the first iteration of the Newton–Raphson algorithm. Typical choices of beta0 include the LASSO or ridge estimates under the cross-sectional phenotype measured at one of the time points or the average of the within-subject phenotypic measurements.

The character string argument func specifies one of the two frameworks (GEE and QIF) to be used for regularized estimation. One of the three working correlations from AR-1, exchangeable, and independence can be called through the input argument corr. For example, corr = “exchangeable,” corr = “AR-1,” and corr = “independence” denote exchangeable, AR-1, and independent correlation, respectively. In addition to the bi-level structure, this package has also included sparsity structures on the group and individual level, respectively. To use the bi-level PQIF under the exchangeable working correlation proposed by Zhou et al. (2022a), we need to specify func = ”QIF,” structure = ”bi-level,” and corr = ”exchangeable” at the same time. It is worthwhile noting that the bi-level selection requires two tuning parameters to impose sparsity. When structure = ”group” or structure = ”individual,” only one of the two tuning parameters lam1 and lam2 is needed.

The Newton–Raphson algorithms implemented in the package *springer* proceed in an iterative manner. The input argument maxits provides the maximum number of iterations determined by the users. We can supply the small positive fraction *ϵ* that is used to ensure the stability of the algorithm through argument tol.

In package *springer*, function cv.springer performs cross-validation based on the regularized coefficients provided by springer. The R code is

cv.springer (clin = NULL,e, g, y, beta0, lambda1, lambda2, nfolds, func, corr, **structure**, maxits = 30,tol = 0.001).

The function cv.springer calls springer to conduct cross-validation over a sequence of tuning parameters and report the corresponding cross-validation error. Therefore, it is not surprising to observe that the two functions share a common group of arguments involving the input of data and specifications on the penalization method used for estimation. Unlike the scalars of lam2 and lam2 in function springer, the arguments lambda1 and lambda2 are user-supplied sequences of tuning parameters. For bi-level selection, cv.springer calculates the prediction error across each pair of tunings determined by lambda1 and lambda2. The number of folds used in cross-validation is specified by nfolds.

### 3.2 Additional supporting functions

Package *springer* also provides multiple supporting functions in addition to the core functions. As MCP is the baseline penalty adopted in all the penalized variable selection methods implemented in the package, the function dmcp denotes its first-order derivative function used in the formulation under the Newton–Raphson algorithm. The function penalty determines the type of sparse structure (individual-, group-, or bi-level) imposed for variable selection. Both the group- and bi-level penalizations involve the empirical norm $\|\eta_{nk}{\|}_{\Sigma_{k}}$ . In practice, the form of Σ_*k*_ is not unique. For example, Σ_*k*_ can be chosen as an identity matrix, and then $\|\eta_{nk}{\|}_{\Sigma_{k}}$ reduces to an L_2_ norm. While the alternatives might be equally applicable, the default choice of Σ_*k*_ in package *Springer* is in the form discussed in Section 2.3.

It is assumed that repeated measurements on the response are given in the wide format with the dimension of 100 by 5, where 100 is the sample size and 5 is the number of time points, then we can use function reformat to convert the wide format to long format with dimension 500 by 1. Similarly, the design matrix under sample size 100 and 50 main and interaction effects has a dimensionality of 100 by 50, if they do not vary across time. Then, reformat will return a 500 by 51 wide format matrix including the column of intercept. An “id” column will also be generated by reformat to show the time points corresponding to 500 columns. Moreover, a simulated dataset, dat, is provided to demonstrate the penalized selection in the proposed longitudinal study. We describe more details in the next section.

## 4 Simulation example

In this section, we demonstrate the fit of bi-level selection using package *Springer* based on simulated datasets. Although model (1) is general in the sense that both the response and predictors are repeatedly measured, it can be reduced to the case where the predictors, consisting of the clinical covariates and environmental and genetic factors, are cross-sectional under the longitudinal response. Model (1) is flexible in which the predictors can have a mixture of cross-sectional and longitudinal measurements. For instance, the repeated measurements are only taken on E factors and not on clinical or G factors.

The motivating dataset for the sparse group variable selection developed in Zhou et al. (2022a) can be retrieved from the Childhood Asthma Management Program (CAMP) in our case study where the clinical, E, and G factors are not repeatedly measured (Childhood Asthma Management Program Research Group, 1999; Childhood Asthma Management Program Research Group Szefler et al., 2000; Covar et al., 2012). Therefore, the current version (version 0.1.7) of package *springer* only accounts for such a case. It is worth noting that technically it is not difficult to extend the package to repeatedly measured predictors because the only difference lies in using time-specific measurements rather than repeating the cross-sectional measurements across all the time points in the estimation procedure. We will discuss potential extensions of the package at the end of this section. In the following simulated example, the longitudinal responses are generated together with cross-sectional predictors. The data-generating function is provided as follows:

Data <- **function** (n,p,k,**q**)

{

y = **matrix** (**rep** (0,n*k),n,k)

sig = matrix (0,p,p)

**for** (i in 1: p) {

**for** (j in 1: p) { sig [i,j] = 0.8^**abs** (i-j) }

}

*# Generate genetic factors*

g = mvrnorm (n,**rep** (0,p),sig)

sig0 = **matrix** (0,**q**,**q**)

**for** (i in 1: **q**) {

**for** (j in 1: **q**) { sig0 [i,j] = 0.8^**abs** (i-j) }

}

*# Generate environmental factors*

e = mvrnorm (n,**rep** (0,**q**),sig0)

E0 = **as.numeric** (g [,1]<=0)

E0 = E0+1

e = **cbind** (E0,e [,-1])

e.out = e

e1 = **cbind** (**rep** (1,**dim**(e)[1]),e)

**for** (i in 1:p) { e = **cbind** (e,g [,i]*e1) }

x = **scale**(e)

ll = 0.3

ul = 0.5

**coef** = **runif** (**q**+25,ll,ul)

**mat** = x [,**c** (1:**q**, (**q**+1), (**q**+2), (**q**+6), (**q**+4), (2*q+2), (2*q+3), (2*q+7),

(2*q+5), (3*q+3), (3*q+4), (3*q+8), (3*q+6),

(4*q+4), (4*q+5), (4*q+9), (4*q+7), (5*q+5),

(5*q+6), (5*q+10), (5*q+8), (6*q+6), (6*q+7),

(6*q+11), (6*q+9), (7*q+7))]

**for** (u in 1:k){ y [,u] = 0.5 + rowSums (coef*mat) }

*#Exchangable correlation for repeated measurements*

sig1 = **matrix** (0,k,k)

**diag** (sig1) = 1

**for** (i in 1: k) {

**for** (j in 1: k) { **if** (j **!**= i){sig1 [i,j] = 0.8} } }

error = mvrnorm (n,**rep** (0,k),sig1)

y = y + error

dat = **list** (y = y,x = x,e = e.out, g = g, **coef** = c (0.5,**coef**))

**return** (dat)

}

In the aforementioned codes, *n*, *p*, and *q* represent the sample size, dimension of the genetic factors, and environmental factors, respectively. The number of repeated measurements is *k*. Now, we simulate a dataset with 400 subjects, 100 G factors, and 5 E factors. The number of repeated measurements is set to 5. The correlation coefficient *ρ* of the compound symmetry working correlation assumed for longitudinal measurements is 0.8. In the data-generating function, coef represents the vector of non-zero coefficients, and mat is the part of design matrix corresponding to the main and interaction effects associated with non-zero coefficients. With (*n*, *p*, *q*) = (400, 100, 5), coef is a vector of length 30, and mat is a 400-by-30 matrix. The R code coef*mat denotes element-wise multiplication by multiplying the non-zero coefficient to the corresponding main or interaction effects. Therefore, rowSums(coef*mat) returns a 400-by-1 vector. The code “0.5 + rowSums(coef*mat)” stand for the combined effects from those important main and interaction effects, and the intercept, with 0.5 being the coefficient multiplied to the intercept. We listed the R codes and output in the following section:

library (MASS)

library (glmnet)

library (springer)

set.seed (123)

n.train = n = 400

*p* = 100; k = 5; q = 5

dat.train = Data(n.train,p,k,q)

y.train = dat.train$y

x.train = dat.train$x

e.train = dat.train$e

g.train = dat.train$g

> dim(y.train)

[1] 400 5

> dim(x.train)

[1] 400 605

> dim(e.train)

[1] 400 5

> dim(g.train)

[1] 400 100

In addition, the R codes dat.train$coef saves the non-zero coefficients used in the data-generating model. By setting the seed, we can reproduce the data generated through calling the Data. A total of 100 genetic factors and 5 environmental factors lead to a total of 605 main and interaction effects, excluding the intercept. We first obtain the initial value of the coefficient vector $\hat{\beta_{0}}$ by fitting ridge regression under the univariate response taken from a single time point. Other choices of initial values include fitting ridge regression or LASSO under the average of within-subject measurements, which accommodate the case of unbalanced data, where a proper single point might be difficult to determine. In general, the regularized estimates remain relatively insensitive to different choices of initial value $\hat{\beta_{0}}$ , as long as $\hat{\beta_{0}}$ is reasonable, in other words, not extremely far away from the optimal solution.

x.train1 = **cbind** (**data**.**frame** (**rep** (1,n)),x.train)

x.train1 = **data**.**matrix** (x.train1)

lasso.cv = cv.glmnet (x.train1,y.train [,1],alpha = 0,nfolds = 5)

alpha = lasso.cv$lambda.**min**/2

lasso.fit = glmnet (x.train1,y.train [,1],

**family** = "gaussian",alpha = 0,nlambda = 100)

beta0 = **as**.**matrix** (**as**.**vector** (**predict** (lasso.fit,

s = alpha, type = "coefficients"))[-1])

With the initial value obtained previously, we call function cv.springer to calculate cross-validation errors corresponding to the pair of tuning parameters (lambda1 and lambda2). The number of fold is 5 by setting nfolds to 5 in the following codes. Then, a penalized bi-level QIF model with an independence correlation has been fitted to the simulated data with the optimal tunings. The fitted regression coefficients are saved in fit.beta.

lambda1 = **seq** (0.025,0.1,**length**.out = 5)

lambda2 = **seq** (1,1.5,**length**.out = 3)

tunning = cv.springer (clin = NULL, e.train, g.train, y.train, beta0,

lambda1, lambda2, nfolds = 5, func = "QIF",

corr = "independence",**structure** = "bilevel",

maxits = 30, tol = 0.1)

lam1 = tunning$lam1

lam2 = tunning$lam2

> lam1

[1] 0.0625

> lam2

[1] 1

> tunning$CV

[,1] [,2] [,3]

[1,] 14.873142 15.37916 16.02844

[2,] 12.282850 13.23239 13.81465

[3,] 9.663655 10.62635 11.96531

[4,] 10.133435 11.00219 12.25365

[5,] 11.237012 11.79566 13.17813

fit.**beta** = springer (clin = NULL, e.train, g.train, y.train, beta0,

func = "QIF",corr = "independence",

**structure** = "bilevel",lam1,lam2,maxits = 30,tol = 0.1)

To assess the model’s performance, we will compare the fitted coefficient vector fit.beta with the true coefficient vector, which is used to simulate the response variable in Data. Since the codes dat.train$coef only report the true non-zero coefficient, the resulting vector has a length much less than fit.beta, which includes zero coefficient. Therefore, we first retrieve locations of non-zero effects in the coefficient vector used to generate the longitudinal response. In the following codes, tp, tp.main, and tp.interaction represent the locations for all the non-zero effects, that is, the column number of the corresponding effects in the design matrix. Although the coefficients are randomly generated from uniform distributions, the locations of the non-zero effects are fixed. In total, there are 30 non-zero effects, consisting of 5 environmental factors, 7 genetic factors, and 18 gene–environment interactions.

## non-zero effects without intercept

tp = **c**(1:**q**, (**q**+1), (**q**+2), (**q**+6), (**q**+4), (2*q+2), (2*q+3), (2*q+7), (2*q+5),

(3*q+3), (3*q+4), (3*q+8), (3*q+6), (4*q+4), (4*q+5), (4*q+9), (4*q+7),

(5*q+5), (5*q+6), (5*q+10), (5*q+8), (6*q+6), (6*q+7), (6*q+11),

(6*q+9), (7*q+7))+1

## non-zero main effects

tp.main = **c**((**q**+2), (2*q+3), (3*q+4), (4*q+5), (5*q+6), (6*q+7), (7*q+8))

## non-zero interaction effects

tp.interaction = **c**((**q**+2), (**q**+6), (**q**+4), (2*q+3), (2*q+7), (2*q+5),

(3*q+4), (3*q+8), (3*q+6), (4*q+5), (4*q+9), (4*q+7), (5*q+6), (5*q+10),

(5*q+8), (6*q+7), (6*q+11), (6*q+9))+1

We run the codes in R console to evaluate the accuracy in parameter estimation. The precision in estimating the regression coefficients has been assessed based on TMSE, MSE, and NMSE, respectively. The mean squared error of the fitted coefficient vector fit.beta with respect to the true one, denoted as TMSE, is defined as

$$\text{TMSE}=\frac{1}{1+p+q+pq}\|{\hat{\beta }}_{n}-\beta_{n}\|,$$

where ${\hat{\beta }}_{n}$ corresponds to fit.beta and *β*_*n*_ is the true regression coefficient vector used to generate the response in the data-generating function. In this simulation example, there are 100 genetic factors (*p* = 100) and 5 environmental factors (*q* = 5), resulting in a coefficient vector of length 606, including the intercept. To observe the estimation accuracy on a finer scale, we further dissect *β*_*n*_ into the component corresponding to tp and calculate the mean square error with respect to the counterpart from fit.beta, denoted as MSE. The mean square error is computed based on the rest of fit.beta, and *β*_*n*_ is defined as NMSE. The R codes and output are listed as follows:

coeff = **matrix** (fit.**beta**, **length** (fit.**beta**),1)

coeff.train = **rep** (0,**length** (coeff))

coeff.train [tp] = dat.train$**coef**[-1]

TMSE = **mean** ((coeff-coeff.train)^2)

MSE = **mean** ((coeff [tp]-coeff.train [tp])^2)

NMSE = **mean** ((coeff [-tp]-coeff.train [-tp])^2)

> TMSE

[1] 0.003455488

> MSE

[1] 0.06563788

> NMSE

[1] 0.0002168221

The dat.train$coef only consists of the non-zero coefficients used to generate longitudinal responses in the data-generating model; therefore, its dimension is not the same as fit.beta as the estimated regression coefficient vector is sparse and includes zero coefficient, thus having a much larger dimension. In regularized variable selection, the non-zero coefficients from fit.beta will not be identical to those in dat.train$coef due to the shrinkage estimation in order to achieve variable selection. The aforementioned output shows the estimation errors in terms of TMSE, MSE, and NMSE, respectively. The NMSE is much smaller than the MSE since it computes the MSE with respect to zero coefficients.

In addition to evaluating the accuracy in parameter estimation, we also examine the performance in identification in terms of number of true- and false-positive effects. Specifically, by comparing the locations of the non-zero components in fit.beta and the true coefficient vector used in the data-generating model, we can report the total number of true- and false-positive effects, such as TP and FP. The identification results have also been summarized for the main genetic effects (TP1 and FP1) and G×E interactions (TP2 and FP2). The locations of important effects saved in tp obtained from the chunk of R codes previously also include the environmental main effects that are not subject to selection. When calculating the number of true and false positives in the next section, we only count the effects that are under selection, corresponding to the 7 G factors and 18 G×E interactions. The output is provided in the following section.

coeff [**abs** (coeff) < 0.1] = 0

coeff [1: (1 + **q**)] = 0

ids = **which** (coeff != 0)

TP = **length** (**intersect** (tp,ids))

res = ids [**is**.**na** (**pmatch** (ids,tp))]

FP = **length** (res)

coeff1 = **rep** (0,**length** (coeff))

coeff1 [1: (1 + **q**)] = coeff [1: (1 + q)]

for (i in (**q**+2):**length** (coeff)) {

**if** ( i%%(**q**+1)==1) coeff1 [i]= coeff[i]

}

ids1 = **which** (coeff1 != 0)

TP1 = **length** (intersect (tp.main,ids1))

res1 = ids1 [**is**.**na** (**pmatch** (ids1,tp.main))]

FP1 = **length** (res1)

coeff2 = coeff

coeff2 [1: (1 + q)] = 0

**for** (i in (**q**+2):**length** (coeff)) {

**if** ( i%%(**q**+1)==1) coeff2[i] = 0

}

ids2 = **which** (coeff2 != 0)

TP2 = **length** (**intersect** (tp.**interaction**,ids2))

res2 = ids2 [**is**.**na** (**pmatch** (ids2,tp.**interaction**))]

FP2 = **length** (res2)

> TP

[1] 21

> FP

[1] 3

> TP1

[1] 6

> FP1

[1] 0

> TP2

[1] 15

> FP2

[1] 3

Results on true and false positives indicate that six out of the seven important main effects have been identified, and 15 out of the 18 interactions used in the data-generating model have been detected. The number of identified false-positive effects is three.

In addition to extensive simulation studies that demonstrate the merit of the proposed sparse group variable selection in longitudinal studies, Zhou et al. (2022a) have also considered scenarios in the presence of missing measurements (Rubin, 1976; Little and Rubin, 2019). Under the pattern of missing completely at random (MCAR), the penalized QIF procedure can still be implemented by using a transformation matrix to accommodate missingness. Such a data-transformation procedure will be incorporated in the release of package *springer* in the near future.

The current version of package *springer* (version 0.1.7) has implemented three working correlation matrices, independence, AR-1, and exchangeable, for individual-, group-, and bi-level variable selection under continuous longitudinal responses in both the GEE and QIF frameworks. The future improvement includes incorporating other working correlations, such as the unstructured working correlation. A question worth exploring is the computational feasibility of unstructured working correlation under QIF as the large number of covariance parameters will potentially lead to much more complicated extended score vectors, incurring prohibitively heavy computational cost for high-dimensional data. We will also consider extensions to discrete responses such as binary, count, and multinomial responses, and longitudinally measured clinical, environmental, and genetic factors, especially after these data are available.

## 5 Case study

We adopt package *springer* to analyze the high-dimensional longitudinal data from the Childhood Asthma Management Program (Childhood Asthma Management Program Research Group, 1999; Childhood Asthma Management Program Research Group Szefler et al., 2000; Covar et al., 2012). Children with age between 5 and 12 years, who are diagnosed with chronic asthma have been included in the study and monitored through follow-up visits over 4 years. The response variable is the forced expiratory volume in one second (FEV1), which indicates the amount of air one can expel from the lungs in one second. We focus on FEV1 that has been repeatedly measured during the 12 visits after the application of treatment ( budesonide, nedocromil, and Control). For our gene–environment interaction analysis, the G factors are the single nucleotide polymorphisms, and E factors consist of treatment, age, and gender. For the demonstration purpose, we target SNPs based on the genes from chromosome 6 and the Wnt signaling pathway at the same time, resulting in a total of 203 SNPs. Following the NIH guideline, we cannot share the data publicly or disclose them in the R output. The data can be applied from dbGap through the accession number phs000166.v2.p1.

# the longitudinal FEV1

> **dim**(ylong)

[1] 438 12

# environmental factors (treatment, age, gender)

> **dim**(e)

[1] 438 3

# genetic factos (SNP)

> **dim**(X)

[1] 438 203

Both the environmental and genetic factors are cross-sectional. For example, as shown previously, each of the three E factors is a 438-by-1-column vector, forming a 438-by-3 matrix. We obtained the optimal tuning parameters using function cv.springer. One can start the process by defining a grid interval for each tuning parameter. We applied the cv.springer function with estimating function type func = ”QIF” and working correlation matrix type corr = ”exchangeable” as follows:

> **library** (springer)

> *#define input arguments*

> lambda1 = **seq** (0.5,1,**length**.out = 5)

> lambda2 = **seq** (3,3.5,**length**.out = 5)

> *#run cross-validation*

> tunning = cv.springer (clin = NULL, e, X, ylong, beta0, lambda1,

+ lambda2, nfolds = 5, func = "QIF”, corr = "exchangeable",

+ **structure** = "bilevel”, maxits = 30, tol = 0.001)

> *#print the results*

> **print** (tuning)

$lam1

[1] 0.5

$lam2

[1] 3

$CV

[,1] [,2] [,3] [,4] [,5]

[1,] 0.2827513 0.2838438 0.2846629 0.2855799 0.2865723

[2,] 0.2858653 0.2867847 0.2877162 0.2885925 0.2894861

[3,] 0.2884425 0.2897974 0.2906588 0.2916546 0.2925146

[4,] 0.2919309 0.2927759 0.2936686 0.2945191 0.2954699

[5,] 0.2948042 0.2954983 0.2962844 0.2971886 0.2979241

The optimal tuning parameters within the range have been selected as 0.5 and 3 for lambda1 and lambda2, respectively. We have then applied the springer function to the dataset using the optimal tuning parameters as follows:

> #fit the bi-level selection model

> **beta** = springer (clin = NULL, e, X, ylong, beta0, func = "QIF",

+ corr = "exchangeable”, **structure** = "bilevel”, lam1, lam2,

+ maxits = 30, tol = 0.001)

The springer function returns the estimated coefficients for the intercept, environmental factors, genetic factors, and G×E interactions. We organized the output to show the identified genetic main effects and G×E interactions in Table 1. The selected SNPs and the corresponding genes are listed in the first two columns. The last four columns contain the estimated coefficients of the main effects for each SNP and the corresponding interactions between the SNPs and environmental factors .

**TABLE 1:** Identified main and interaction effects based on the genes from the Wnt signaling pathway on chromosome 6.

SNP	Gene		Treatment	Age	Gender
rs10948011	TAF8	0	0	0	−0.020
rs33954419	USP49	−0.012	0	0	0
rs12194513	TAF8	0.005	0	0	0
rs205339	MAP3K7	0.016	0	0	0
rs11970772	CCND3	0	0.102	0	0.069
rs1018155	DAAM2	0	0	−0.169	0
rs913574	DAAM2	0	−0.020	0	0
rs13191407	MAP3K7	0	0	−0.009	−0.023
rs2475802	MOCS1	0.095	0	0	0
rs805300	BAG6	−0.110	0	0	0
rs1475114	MOCS1	−0.047	0	0	0
rs1018156	DAAM2	−0.045	0	0	0
rs4607417	CCND3	0	−0.108	0	0
rs284513	MAP3K7	0	0.040	0.075	0.011
rs17812916	RSPO3	0	0.021	0	0.208
rs2077102	BAG6	0	0	−0.266	−0.016
rs3218100	CCND3	0.003	0	0	0
rs2242655	C6orf47	−0.046	0	0	0
rs2493835	TAF8	0.056	0	0	0
rs9491700	RSPO3	0.009	0	0	0
rs3008819	MOCS1	−0.021	0	0	0
rs2255741	PRRC2A	0.066	−0.021	0	0
rs3003931	DAAM2	0.004	0	0	0
rs791048	MAP3K7	0	0.080	0	0
rs9285458	RSPO3	0	0	−0.049	−0.078
rs3008801	DAAM2	−0.072	0	0	0
rs9462082	PPARD	0.026	0	0	0
rs166920	MAP3K7	−0.009	0	0	0
rs1144159	MAP3K7	0.091	0	0	0
rs284512	MAP3K7	0	−0.101	0	0
rs719726	RSPO3	0	−0.028	0.020	0.130
rs6916203	DAAM2	0	0	0	0.010
rs2504097	DAAM2	0	0	0	−0.034
rs4713858	FANCE	0	0	−0.139	0.157
rs1936789	RSPO3	0	−0.030	−0.044	0.072
rs1923084	MAP3K7	0	−0.163	0	0.315
rs9462769	C6orf132	0	0	−0.094	−0.138
rs11759168	DAAM2	0.173	0.027	0	−0.174
rs707917	ABHD16A	−0.096	0	0.196	0.001
rs9267531	CSNK2B	−0.141	0	0	0
rs9394630	DAAM2	0.116	0	0	0
rs2504790	DAAM2	−0.133	0	0	0
rs2750456	MAP3K7	−0.052	0	0	0
rs3003933	DAAM2	−0.073	0	0	0
rs2984659	MOCS1	0.004	0	0	0
rs282065	MAP3K7	0.076	0	0	0
rs2504805	DAAM2	0	0	0	0.122
rs1046080	PRRC2A	0	0	−0.184	0

## 6 Discussion

Before the formulation of the bi-level (or sparse group) selection in high-dimensional statistics (Friedman et al., 2010b), the relevant statistical models have already been extensively studied in genetic association studies (Lewis, 2002; Wu et al., 2012), which involve the simultaneous selection of important pathways (or gene sets) and corresponding genes within the pathways (or gene sets) (Schaid et al., 2012; Wu and Cui, 2014; Jiang et al., 2017). For G×E interaction studies, the bi-level selection has served as the umbrella model and led to a wide array of extensions (Zhou et al., 2021a).

Package *springer* cannot be applied directly on the ultra-high-dimensional data (Fan and Lv, 2008), which is essentially due to the limitation of regularization methods. A more viable path is to conduct marginal screening first and then apply regularization methods on a smaller set of features suitable for penalized selection (Jiang et al., 2015; Li et al., 2015; Wu et al., 2019). In fact, such an idea on screening has motivated the migration of joint analyses to marginal penalization in recent G×E studies (Chai et al., 2017; Lu et al., 2021; Wang et al., 2022). It is marginal in the sense that only the main and interaction effects with respect to the same G factor are considered in the model. Thus, marginal penalization is of a parallel nature and suitable for handling the ultra-high-dimensional data. To use our R package conducting marginal regularization on the ultra-high-dimensional longitudinal data, we just need to set the argument g in function springer to one genetic factor at a time, which will return the regression coefficients for all the clinical and environmental factors and main G and G×E interactions with respect to that G factor. The magnitude of the coefficients corresponding to the effects subject to the selection will be used as the measure for ranking and selecting important effects.

Robust penalization methods have drawn increasing attention in recent years (Freue et al., 2019; Hu et al., 2021; Chen et al., 2022; Sun et al., 2022). In high-dimensional longitudinal studies, incorporation of robustness is more challenging. The corresponding variable selection methods are expected to be insensitive to not only the outliers and data contaminations but also to misspecification of working correlation structure capturing the correlations among repeated measurements. It has been widely recognized that GEE is vulnerable to long-tailed distributions in the response variable, even though it yields consistent estimates when working correlations are misspecified (Qu and Song, 2004). Therefore, the more robust QIF emerges as a powerful alternative for developing variable selection methods. Our R package *springer* can facilitate further understanding of robustness in bi-level selection models.

## Data Availability

The original contributions presented in the study are included in the article/Supplementary Material; further inquiries can be directed to the corresponding author. Authorized access should be granted before accessing the data analyzed in the case study. Request to access the data should be sent to Database of Genotype and Phenotype (dbGaP) at https://www.ncbi.nlm.nih.gov/projects/gap/cgi-bin/study.cgi?study_id=phs000166.v2.p1 through accession number phs000166.v2.p1.
