# Supplementary material for: Springer: An R package for bi-level variable selection of high-dimensional longitudinal data
Source: Front Genet. 2023 Apr 6;14:1088223. doi: 10.3389/fgene.2023.1088223 (PMC10117642; doi:10.3389/fgene.2023.1088223)
Supplement: Supplementary file 1 [file DataSheet1.pdf]

Supplementary Material for “Springer: An R Package for bi-level variable selection of high-dimensional longitudinal data ” by Fei Zhou, Yuwen Liu, Jie Ren, Weiqun Wang and Cen Wu

Appendix

A Derivations of Alternative Methods based on QIF

In Package *springer*, there are two types of alternative methods within the QIF framework, the penalized group QIF that ignores the within group sparsity and conducts selection only on the group level, and the penalized QIF that perform regularized selection individually without considering a group structure.

A.1 Penalized Group QIF

The penalized group QIF implemented in package *springer* has the score equation defined below:

$$U(\beta_n) = Q(\beta_n) + \sum_{k=1}^{p} \rho(||\eta_{nk}||_{\Sigma_k}; \lambda_1, \gamma),$$

where ρ denotes the minimax concave penalty with tuning parameters λ_1 and γ. Based on Section 2.2, the coefficient vector β_n represents all the main and interaction effects. $\eta_n k$ denotes the $(q+1)$ main genetic and G$\times$E interaction effects with respect to the kth G factor. Regularized identification has been only conducted on the group level because the shrinkage is imposed on $||\eta_{nk}||_{\Sigma_k}$, the empirical norm of η_k.

Again, the Newton-Raphson algorithm can facilitate model fitting to obtain $\hat{\beta}_n$, the penalized QIF estimate. At the $(g+1)$th iteration, we solve for $\hat{\beta}_n^{g+1}$ using $\hat{\beta}_n^{g}$ from the previous iteration as follows:

$$\hat{\beta}_n^{g+1} = \hat{\beta}_n^{g} + [V(\hat{\beta}_n^{g}) + nH(\hat{\beta}_n^{g})]^{-1}[P(\hat{\beta}_n^{g}) - nH(\hat{\beta}_n^{g})\hat{\beta}_n^{(g)}],$$

with $P(\hat{\beta}_n^{g})$ and $V(\hat{\beta}_n^{g})$ being defined as:

$$P(\hat{\beta}_n^{g}) = -\frac{\partial Q((\hat{\beta}_n^{g}))}{\partial \beta_n} = -2\frac{\partial \overline{\phi_n}^\top}{\partial \beta_n}\overline{\Omega_n}^{-1}\overline{\phi_n}(\hat{\beta}_n^{g}),$$

$$V(\hat{\beta}_n^{g}) = \frac{\partial^2 Q(\hat{\beta}_n^{(g)})}{\partial^2 \beta_n} = 2\frac{\partial \overline{\phi_n}^\top}{\partial \beta_n}\overline{\Omega_n}^{-1}\frac{\partial \overline{\phi_n}}{\partial \beta_n}.$$

In addition, $H(\hat{\beta}_n^g)$ is a diagonal matrix with the derivatives of MCP being the diagonal elements as follows:

$$H(\hat{\beta}_n^g) = \text{diag}(\underbrace{0, ..., 0}_{1+q}, \underbrace{\frac{\rho'(||\hat{\eta}_{n1}^g||_{\Sigma_1}; \lambda_1, \gamma)}{\epsilon + ||\hat{\eta}_{n1}^g||_{\Sigma_1}}, ..., \frac{\rho'(||\hat{\eta}_1^g||_{\Sigma_1}; \lambda_1, \gamma)}{\epsilon + ||\hat{\eta}_{n1}^g||_{\Sigma_1}}}_{1+q}, ...,$$
$$\underbrace{\frac{\rho'(||\hat{\eta}_p^{(g)}||_{\Sigma_p}; \lambda_1, \gamma)}{\epsilon + ||\hat{\eta}_{np}^g||_{\Sigma_p}}, ..., \frac{\rho'(||\hat{\eta}_{np}^g||_{\Sigma_p}; \lambda_1, \gamma)}{\epsilon + ||\hat{\eta}_{np}^g||_{\Sigma_p}}}_{1+q}),$$

where λ_1 is the tuning parameter controlling the sparsity of the group level effects, and γ is the regularization parameter determining the concavity of MCP. In Section 2.4, a factor of $\sqrt{q+1}$ is multiplied to λ_1 so the group size can be adjusted in bi-level selection. With only the group level penalty, it is no longer necessary because all the groups are of the same size.

We set the first $(q+1)$ entries on the main diagonal of $H(\hat{\beta}_n^g)$ to zero, since the intercept and main environmental effects are not subject to selection. The first and second derivative functions of group MCP can be approximated by $nH(\hat{\beta}_n^g)$ and $nH(\hat{\beta}_n^g)\hat{\beta}_n^g$, respectively. The iterative Newton algorithm proceeds and $\hat{\beta}_n^g$ can be updated across iterations. The stopping criterion is the same as we have adopted for the bi-level QIF. That is, the difference between two penalized estimates from successive iterations is small in terms of L1 norm. In general, convergence can be achieved in a few iterations.

A.2 Penalized QIF

Penalized QIF conducts selection of main and interaction effects in the G×E studies without accounting for the group structure. The penalized score function can be expressed as:

$$U(\beta_n) = Q(\beta_n) + \sum_{k=1}^{p} \sum_{h=1}^{q+1} \rho(|\eta_{nhk}|; \lambda_1, \gamma),$$

where regression coefficient η_{nhk} denotes the kth element of η_{nk}. The Newton-Raphson update of $\hat{\beta}_n$ is derived as:

$$\hat{\beta}_n^{g+1} = \hat{\beta}_n^g + [V(\hat{\beta}_n^g) + nH(\hat{\beta}_n^g)]^{-1}[P(\hat{\beta}_n^g) - nH(\hat{\beta}_n^g)\hat{\beta}_n^{(g)}],$$

where $P(\hat{\beta}_n^g)$ and $V(\hat{\beta}_n^g)$ are defined as follows:

$$P(\hat{\beta}_n^g) = -\frac{\partial Q((\hat{\beta}_n^g))}{\partial \beta_n} = -2\frac{\partial \overline{\phi_n}^\top}{\partial \beta_n} \overline{\Omega_n}^{-1} \overline{\phi_n}(\hat{\beta}_n^g),$$

$$V(\hat{\beta}_n^g) = \frac{\partial^2 Q(\hat{\beta}_n^{(g)})}{\partial^2 \beta_n} = 2\frac{\partial \overline{\phi_n}^\top}{\partial \beta_n} \overline{\Omega_n}^{-1} \frac{\partial \overline{\phi_n}}{\partial \beta_n}.$$

The main diagonal of $H(\hat{\beta}_n^g)$ includes the first order derivative of MCP:

$$H(\hat{\beta}_n^g) = \text{diag}(\underbrace{0, ..., 0}_{1+q}, \underbrace{\frac{\rho'(|\hat{\eta}_{n11}^g|; \lambda_1, \gamma)}{\epsilon + |\hat{\eta}_{n11}^g|}, ..., \frac{\rho'(|\hat{\eta}_{n1(q+1)}^g|; \lambda_1, \gamma)}{\epsilon + |\hat{\eta}_{n1(q+1)}^g|}}_{1+q}, ...,$$

$$\underbrace{\frac{\rho'(|\hat{\eta}_{np1}^g|; \lambda_1, \gamma)}{\epsilon + |\hat{\eta}_{np1}^g|}, ..., \frac{\rho'(|\hat{\eta}_{np(q+1)}^g|; \lambda_1, \gamma)}{\epsilon + |\hat{\eta}_{np(q+1)}^g|}}_{1+q}),$$

where λ_1 and γ are the tuning and regularization parameters, respectively. The first $q+1$ zeros on the main diagonal of $H(\hat{\beta}_n^g)$ leave no shrinkage on the coefficients corresponding to the intercept and E factors. The Newton algorithm proceeds in a similar manner as fitting for the bi-level and group level models.

B Derivations of Alternative Methods based on GEE

In addition to penalized QIF methods, package *springer* also provides the individual-, group- and bi-level feature selection based on GEE. We briefly introduce these counterparts of the aforementioned penalized QIF methods below.

B.1 Penalized Bi-level GEE

$$\tilde{U}(\beta_n) = \tilde{Q}(\beta_n) - \sum_{k=1}^{p} \rho'(||\eta_{nk}||_{\Sigma_k}; \lambda_1, \gamma) - \sum_{k=1}^{p}\sum_{h=1}^{q+1} \rho'(|\eta_{nkh}|; \lambda_2, \gamma)\text{sgn}(\eta_{nkh}),$$

where the first order derivative of MCP, $\rho'(\cdot)$, has been placed on both the empirical group norm $||\eta_{nk}||_{\Sigma_k}$, and η_{nkh} that represents the individual level effects. Here, $\tilde{Q}(\beta_n)$ is the score equation of GEE, defined as $\tilde{Q}(\beta_n) = \sum_{i=1}^{n} W_i^T V_i^{-1}(Y_i - \mu_i(\beta_n))$. We refer the readers to Section 2.2 where the rationale of developing the above sparse group penalty in longitudinal G×E interaction studies has been described in details.

In general, the Newton-Raphson algorithm can be applied to the optimization problem outlined above. The iterative update of $\hat{\beta}_n$ at the $(g+1)$th iteration can be derived as:

$$\hat{\beta}_n^{g+1} = \hat{\beta}_n^g + [\tilde{V}(\hat{\beta}_n^g) + n\tilde{H}(\hat{\beta}_n^g)]^{-1}[\tilde{Q}(\hat{\beta}_n^g) - n\tilde{H}(\hat{\beta}_n^g)\hat{\beta}_n^g],$$

where $\tilde{V}(\hat{\beta}_n^g)$ denotes the first order derivative of $-\tilde{Q}(\beta_n)$. Besides, the diagonal matrix $H(\hat{\beta}_n^g)$ corresponds to the effects from both the individual– and group– level penalty functions, that

is,

$$\tilde{H}(\hat{\beta}_n^g) = \text{diag}(\underbrace{0, ..., 0}_{1+q}, \underbrace{\frac{\rho'(||\hat{\eta}_{n1}^g||_{\Sigma_1}; \sqrt{q+1}\lambda_1, \gamma)}{\epsilon + ||\hat{\eta}_{n1}^g||_{\Sigma_1}}, ..., \frac{\rho'(||\hat{\eta}_{n1}^g||_{\Sigma_1}; \sqrt{q+1}\lambda_1, \gamma)}{\epsilon + ||\hat{\eta}_{n1}^g||_{\Sigma_1}}}_{1+q}, ...,$$

$$\underbrace{\frac{\rho'(||\hat{\eta}_{np}^g||_{\Sigma_p}; \sqrt{q+1}\lambda_1, \gamma)}{\epsilon + ||\hat{\eta}_{np}^g||_{\Sigma_p}}, ..., \frac{\rho'(||\hat{\eta}_{np}^g||_{\Sigma_p}; \sqrt{q+1}\lambda_1, \gamma)}{\epsilon + ||\hat{\eta}_{np}^g||_{\Sigma_p}}}_{1+q}) + \text{diag}(\underbrace{0, ..., 0}_{1+q},$$

$$\underbrace{\frac{\rho'(|\hat{\eta}_{n11}^g|; \lambda_2, \gamma)}{\epsilon + |\hat{\eta}_{n11}^g|}, ..., \frac{\rho'(|\hat{\eta}_{n1(q+1)}^g|; \lambda_2, \gamma)}{\epsilon + |\hat{\eta}_{n1(q+1)}^g|}}_{1+q}, ..., \underbrace{\frac{\rho'(|\hat{\eta}_{np1}^g|; \lambda_2, \gamma)}{\epsilon + |\hat{\eta}_{np1}^g|}, ..., \frac{\rho'(|\hat{\eta}_{np(q+1)}^g|; \lambda_2, \gamma)}{\epsilon + |\hat{\eta}_{np(q+1)}^g|}}_{1+q}),$$

where $\tilde{H}(\hat{\beta}_n^g)$ is the sum of two diagonal matrices including the derivatives of MCP and group MCP on the main diagonal, respectively. The first $(1+q)$ entries on the main diagonal are zeros, indicating the intercept and environmental main effects are not subjected to penalized selection.

The iterative update of $\hat{\beta}_n$ terminates when the L_1 difference between $\hat{\beta}_n^{g+1}$ and $\hat{\beta}_n^g$ is smaller than a predefined threshold. In R package *springer*, we have adopted the same stopping criterion for all the implemented methods in both the GEE and QIF framework.

B.2 Penalized Group GEE

The penalized group QIF implemented in package *springer* has the score equation defined below:

$$\tilde{U}(\beta_n) = \tilde{Q}(\beta_n) - \sum_{k=1}^{p} \rho'(||\eta_{nk}||_{\Sigma_k}; \lambda_1, \gamma),$$

where $\rho'(\cdot)$ is the first order derivative of the minimax concave penalty, and we define the score equation of GEE as $\tilde{Q}(\beta_n) = \sum_{i=1}^n W_i^T V_i^{-1}(Y_i - \mu_i(\beta_n))$. The coefficient vector β_n corresponds to all the main and interaction effects, and for the kth genetic factor, η_{nk} represents the $(q+1)$ main genetic and G×E interaction effects. The group level shrinkage has been imposed on $||\eta_{nk}||_{\Sigma_k}$, the empirical norm of η_k.

At the $(g+1)$th iteration of the the Newton-Raphson algorithm, we solve for $\hat{\beta}_n^{g+1}$ using $\hat{\beta}_n^g$ from the previous iteration as follows:

$$\hat{\beta}_n^{g+1} = \hat{\beta}_n^g + [\tilde{V}(\hat{\beta}_n^g) + n\tilde{H}(\hat{\beta}_n^g)]^{-1}[\tilde{Q}(\hat{\beta}_n^g) - n\tilde{H}(\hat{\beta}_n^g)\hat{\beta}_n^{(g)}],$$

with $\tilde{V}(\hat{\beta}_n^g)$ being the first order derivative of $-\tilde{Q}(\hat{\beta}_n^g)$. The diagonal matrix $\tilde{H}(\hat{\beta}_n^g)$ is defined as:

$$\tilde{H}(\hat{\beta}_n^g) = \text{diag}(\underbrace{0, ..., 0}_{1+q}, \underbrace{\frac{\rho'(||\hat{\eta}_{n1}^g||_{\Sigma_1}; \lambda_1, \gamma)}{\epsilon + ||\hat{\eta}_{n1}^g||_{\Sigma_1}}, ..., \frac{\rho'(||\hat{\eta}_1^g||_{\Sigma_1}; \lambda_1, \gamma)}{\epsilon + ||\hat{\eta}_{n1}^g||_{\Sigma_1}}}_{1+q}, ...,$$

$$\underbrace{\frac{\rho'(||\hat{\eta}_p^{(g)}||_{\Sigma_p}; \lambda_1, \gamma)}{\epsilon + ||\hat{\eta}_{np}^g||_{\Sigma_p}}, ..., \frac{\rho'(||\hat{\eta}_{np}^g||_{\Sigma_p}; \lambda_1, \gamma)}{\epsilon + ||\hat{\eta}_{np}^g||_{\Sigma_p}}}_{1+q}),$$

where the first $(q+1)$ entries on the main diagonal are zeros corresponding the main effects not subject to selection, and the rest nonzero entries are the first order derivatives of group MCP, corresponding to the kth G factor, respectively. Therefore, $nH(\hat{\beta}_n^g)\hat{\beta}_n^g$ denotes the second order derivative function of group MCP in the aforementioned Newton-Raphson update using $\hat{\beta}_n^g$. In $\tilde{H}(\hat{\beta}_n^g)$, λ_1 and γ are the tuning and regularization parameters determining the magnitude of group level shrinkage and the concavity of group MCP, correspondingly. Since the group size is a constant, it is not necessary to adjust the group size by multiplying a factor of $\sqrt{q+1}$ to the group norm as in the bi-level selection case.

B.3 Penalized GEE

Penalized GEE selects important main and interaction effects in the longitudinal G×E studies. The penalized GEE function can be expressed as:

$$\tilde{U}(\beta_n) = \tilde{Q}(\beta_n) - \sum_{k=1}^{p}\sum_{h=1}^{q+1} \rho'(|\eta_{nhk}|; \lambda_1, \gamma)\text{sgn}(\eta_{nhk}),$$

where the score equation of GEE is denoted as $\tilde{Q}(\beta_n) = \sum_{i=1}^{n} W_i^T V_i^{-1}(Y_i - \mu_i(\beta_n))$, and $\rho'(\cdot)$ is the first order derivative of MCP. The Newton-Raphson algorithm leads to the following iterative update of $\hat{\beta}_n$:

$$\hat{\beta}_n^{g+1} = \hat{\beta}_n^g + [\tilde{V}(\hat{\beta}_n^g) + n\tilde{H}(\hat{\beta}_n^g)]^{-1}[\tilde{Q}(\hat{\beta}_n^g) - n\tilde{H}(\hat{\beta}_n^g)\hat{\beta}_n^{(g)}],$$

where $\tilde{V}(\hat{\beta}_n^g)$ is the first order derivatives of $-\tilde{Q}(\beta_n)$. The diagonal matrix $\tilde{H}(\hat{\beta}_n^g)$ is defined as follows:

$$\tilde{H}(\hat{\beta}_n^g) = \text{diag}(\underbrace{0, ..., 0}_{1+q}, \underbrace{\frac{\rho'(|\hat{\eta}_{n11}^g|; \lambda_1, \gamma)}{\epsilon + |\hat{\eta}_{n11}^g|}, ..., \frac{\rho'(|\hat{\eta}_{n1(q+1)}^g|; \lambda_1, \gamma)}{\epsilon + |\hat{\eta}_{n1(q+1)}^g|}}_{1+q}, ...,$$
$$\underbrace{\frac{\rho'(|\hat{\eta}_{np1}^g|; \lambda_1, \gamma)}{\epsilon + |\hat{\eta}_{np1}^g|}, ..., \frac{\rho'(|\hat{\eta}_{np(q+1)}^g|; \lambda_1, \gamma)}{\epsilon + |\hat{\eta}_{np(q+1)}^g|}}_{1+q}),$$

where the first q+1 zero entries correspond to the environmental main effects that are not under penalized selection, the rest $p(q+1)$ entries are the first order derivative of MCP with respect to the main genetic effects as well as G×E interactions. In the expression of $\tilde{H}(\hat{\beta}_n^g)$, the tuning and regularization parameters are denoted as λ_1 and γ, separately.
